# Factors Affecting the Welfare of Unweaned Dairy Calves Destined for Early Slaughter and Abattoir Animal-Based Indicators Reflecting Their Welfare On-Farm

**DOI:** 10.3389/fvets.2021.645537

**Published:** 2021-04-16

**Authors:** Laura A. Boyle, John F. Mee

**Affiliations:** Teagasc, Animal and Grassland Research and Innovation Centre, Fermoy, Ireland

**Keywords:** bull calf, welfare indicators, ante- mortem, post-mortem, meat inspection, health, pasture-based, slaughter

## Abstract

In many dairy industries, but particularly those that are pasture-based and have seasonal calving, “surplus calves,” which are mostly male, are killed at a young age because they are of low value and it is not economically viable to raise them. Such calves are either killed on farm soon after birth or sent for slaughter at an abattoir. In countries where calves are sent for slaughter the age ranges from 3-4 days (New Zealand and Australia; “bobby calves”) to 3-4 weeks (e.g., Ireland); they are not weaned. All calves are at the greatest risk of death in the 1st month of life but when combined with their low value, this makes surplus calves destined for early slaughter (i.e., <1 month of age) particularly vulnerable to poor welfare while on-farm. The welfare of these calves may also be compromised during transport and transit through markets and at the abattoir. There is growing recognition that feedback to farmers of results from animal-based indicators (ABI) of welfare (including health) collected prior to and after slaughter can protect animal welfare. Hence, the risk factors for poor on-farm, in-transit and at-abattoir calf welfare combined with an ante and post mortem (AM/PM) welfare assessment scheme specific to calves <1 month of age are outlined. This scheme would also provide an evidence base with which to identify farms on which such animals are more at risk of poor welfare. The following ABIs, at individual or batch level, are proposed: AM indicators include assessment of age (umbilical maturity), nutritional status (body condition, dehydration), behavioral status (general demeanor, posture, able to and stability while standing and moving, shivering, vocalizations, oral behaviors/cross-sucking, fearfulness, playing), and evidence of disease processes (locomotory ability [lameness], cleanliness/fecal soiling [scour], injuries hairless patches, swellings, wounds], dyspnoea/coughing, nasal/ocular discharge, navel swelling/discharge); PM measures include assessment of feeding adequacy (abomasal contents, milk in rumen, visceral fat reserves) and evidence of disease processes (omphalitis, GIT disorders, peritonitis, abscesses [internal and external], arthritis, septicaemia, and pneumonia). Based on similar models in other species, this information can be used in a positive feedback loop not only to protect and improve calf welfare but also to inform on-farm calf welfare management plans, support industry claims regarding animal welfare and benchmark welfare performance nationally and internationally.

## Introduction

In dairy industries worldwide, the focus on milk production means that male calves are surplus to requirements. Some female calves are also surplus to requirements as 60% of the milking herd can produce a sufficient number of replacement females (1). The fate of these surplus calves varies between countries depending on the system of production (calving pattern, breed used etc.) the calf price and consumer preference for veal [slaughter age 5–11 months, (2)] or beef [slaughter age >12 months, (2)] (3). The variation is such that in Germany, surplus calves from dual-purpose dairy cows are raised for beef (4) while in the Netherlands, France and Italy, the veal industry is the major outlet [(5) cited by (3)]. This is also the case for most calves produced in North America though calves are also killed soon after birth (6).

A recent review of animal welfare in pasture-based systems of milk production concluded that farm management is as important as the system of management (7), a concept which applies particularly to calf management. Indeed the early (<1 month old) slaughter of surplus calves is particularly associated with pasture-based systems of milk production, where calving is usually seasonal to match the start of grass growth. In such temperate dairying regions, the majority of calves are born in spring (northern hemisphere, e.g., Ireland) or in the autumn (southern hemisphere, e.g., New Zealand and Australia). Furthermore, calving often occurs over a very short timeframe of approximately 8 weeks where a “compact calving” pattern optimizes profitability of the system (8). The seasonality of calf births means that pasture-based production systems are generally incompatible with a veal industry based in the same country, as veal production relies on calf availability all-year-round. This means that such milk production systems face a particular challenge in finding an outlet for surplus calves. The main outlets are that calves are (1) reared for beef in the country of origin, (2) exported to a veal or intensive beef industry in another country or (3) they are killed early, either on-farm soon after birth or slaughtered at a licensed premises within 1 month of birth—(3). For slaughter calves, the age ranges from 3–4 days (New Zealand and Australia; “bobby calves”) to 3–4 weeks (Ireland, other EU countries and the UK).

Ireland is an example of a country with an intensive pasture-based system of milk production with a seasonal calving pattern as described above. About 40% of all calves born in Irish dairy herds are reared for beef primarily in pasture-based systems of production, while almost 12% of dairy calves 180 k dairy calves [predominately male–out of approximately 1.6 m dairy cows; (9)] are shipped unweaned to the European continent for veal [or beef—(10)] production. Export of calves to a second country is a contentious practice with opponents arguing not to transport young unweaned animals over long distances because it poses major threats to their welfare (11). Indeed, there is scientific evidence to support the detrimental impact of long distance travel on the health and welfare of cattle of all ages (12, 13). In addition, Knowles et al. (14) reported that calves under 1 month of age are physiologically unable to adapt and therefore to cope with transport. The age at which the calves are transported (c. 3 weeks old) coincides with the decrease in maternal antibody and the immaturity of the humoral immune system (15) leaving them more susceptible to environmental infections. There are also concerns with calves' fate at the destination, particularly with veal, but also with intensive beef production, relating to feeding and housing practices and associated antimicrobial use (3, 10, 16). There is considerable room for improvement to calf transport and veal production systems and such changes might make these options for surplus calves more sustainable and ethically acceptable from a societal point of view (3).

In contrast, the ethical issues surrounding slaughter of unweaned calves are such that it is never likely to be acceptable to society (17). One of the most promising potential solutions to surplus calves in general involves use of sexed semen either to ensure the production of female replacements when using dairy genetics or to produce males from beef sires (18–20). Indigenous (rose) veal industries and systems in which calves stay with their dams could also play a role in reducing the need to slaughter calves (3). However, none represents a panacea and several are associated with considerable research gaps (21) as well as political and economic constraints. Hence, it is likely that slaughter of unweaned, mostly male, surplus calves will continue for some time. Given their young age as well as the societal and retail focus on this cohort of animals [e.g., (22)] it is crucial to protect their welfare during their short lives. The vulnerability of these animals to poor welfare was highlighted by a scandal involving cruel treatment of “bobby calves” in New Zealand in 2015 (23). Subsequently, legislation was passed to better protect bobby calves by ensuring a maximum duration of travel of 12 h (from August 2016) and that calves were slaughtered within 12 h of last feed (from Feb 2017) (22). Accordingly, the mortality rate at the abattoir prior to slaughter declined in New Zealand [(24, 25) cited by (26)]. This decline in pre-slaughter mortality rate also reflected education and extension efforts by various industry stakeholders in the New Zealand dairy industry (26). One such successful dairy industry initiative involved a checklist to assist farmers and hauliers in decision making regarding calves fitness for transport. Checks relating to age (minimum of 4 days old), ability to stand, brightness/alertness of eyes and ears, presence of an ear tag, dryness of the navel, hoof hardness, fullness of the stomach and absence of scour (27). Such an animal-based welfare assessment is in line with recommendations of the European Food Safety Authority (EFSA) Panel on Animal Health and Welfare (28).

Ideally, regular comprehensive on-farm welfare assessments would help to protect the welfare of surplus calves while on-farm. However, such assessments are labor-intensive, time-consuming and run the risk of facilitating disease transmission (29). Many conditions that compromise calf welfare that occur on-farm can be assessed at the abattoir (30). Velarde and Dalmau (31) describe a Welfare Quality^®^ assessment for pigs and cattle at the slaughterhouse. Such abattoir-based welfare assessments are not necessarily for use in routine veterinary surveillance (32). Therefore, there are recommendations to incorporate welfare indicators during meat inspection at abattoirs as a voluntary monitoring tool for animal health and welfare (33, 34).

EFSA recommends that animal-based indicators (ABI) be used when assessing welfare in the slaughterhouse (28). ABIs also inform on pre-slaughter handling and transport practices [e.g., (35)]. They are the most valid method of assessing animal welfare because the assessments are of the animals themselves, not their resources, which facilitates comparisons across all systems of husbandry (36). In recent years, numerous studies investigated ABI prior to (ante) and post (mortem) [AM/PM] slaughter in various species [(37–39) [pigs]; (40) [sheep], (41) [cull cows], (42) [cattle]]. Some authors validated specific PM ABIs as indicators of pig health and welfare on-farm [e.g., pig carcass tail lesions (34) and lung pathologies (43)]. Similarly, research on ABI in calves in the slaughterhouse focused on veal calves where PM evaluation of lung pathologies complemented on farm welfare assessments [e.g., (44, 45)]. While the value of necropsy findings from unweaned calves that die on farm is recognized [e.g., (46)] there are only two studies which looked at ABI PM in unweaned calves slaughtered early (26, 47). Both studies involved bobby calves as they were based in New Zealand. Meanwhile, a report of the New Zealand Ministry for Primary Industries (48) presented a systematic mapping review of ABI that could be used to assess the welfare of bobby calves in lairage at commercial abattoirs but these did not include ABI for measurement PM.

Currently, the only routinely collected data relating to the welfare of surplus, unweaned calves are mortality rate on-farm and pre-slaughter (dead or condemned/euthanised at the abattoir). Presumed cause of death, based on PM examination, in bobby calves that die or are condemned prior to slaughter is also recorded in New Zealand. We propose that standardized protocols to record ABI in the slaughterhouse could help to protect the welfare of surplus, unweaned calves destined for early slaughter.

Our main aim in this paper is to identify AM/PM ABI potentially relevant to the welfare (and health) of unweaned dairy calves (<1 month old) on-farm and in transit. This paper frames the issue of slaughtering unweaned calves around pasture-based dairy production systems with seasonal calving patterns such as in Ireland and New Zealand. However, the AM/PM scheme proposed herein could easily be adopted to other production systems disposing of surplus unweaned calves by means of slaughter. Further, we draw on findings from studies reporting slaughterhouse findings in unweaned, mostly “bobby,” calves, studies relating market/auction (<1 month of age) and abattoir (<12 months)-based ABI findings in veal calves and PM findings for dairy calves that die on farm at all ages. As such, this concept could be extended to the slaughter of young cattle in general. Additionally, we outline how a calf AM/PM scheme could work in practice, elaborate on the associated benefits to dairy industries and make recommendations for research in this area. We also cite literature emanating from all production systems on calf mortality, and aspects of calf management with a focus on sex differences, of relevance to surplus, unweaned, calves destined for early slaughter. Our approach is narrative, this is not a systematic review. Hence, we marshall relevant literature to build the case for the need for an AM/PM scheme whereby salient papers are cited but other similar papers may not be.

## On-Farm Management and Mortality Rates of Unweaned Calves Destined for Slaughter in Seasonally Calving Pasture-Based Systems

### Ireland

Abolition of the European Union milk quota brought about significant sectoral changes in the dairy industries of member states. Ireland was one of the countries that saw the greatest increase in the size of the national herd (49). Larger herds means more calves born on dairy farms and recent research indicates that this is positively associated with the probability that calves are slaughtered early (50). Hence, expansion results in an increase in the number of unweaned calves sent for slaughter. In Ireland, expansion also resulted in a renewed focus on breeding for milk production characteristics (51). This combined with an initial increase in the proportion of producers using Jersey genetics (52). These breeding-related changes resulted in an increase in the number of dairy male calves with low beef, and at the extreme, no veal, characteristics, and therefore of very low economic value. Hence, in recent years Irish Animal Identification and Movement (AIM) bovine statistics indicate that a proportionately small number (c. 30 k in 2019) of predominately-male, unweaned dairy calves are slaughtered in Ireland each year [e.g., (9)]. Under EU legislation (Council Regulation (EC) 1/2005) it is illegal to transport calves <10 days old over distances >100 km in the EU so while these animals appear in the 0–6week old category in the AIM bovine statistics (9) they are generally 3–4 weeks old at slaughter.

### New Zealand and Australia

In New Zealand and Australia, there are no opportunities to rear or to export surplus or “bobby” calves for veal. These calves are killed on-farm by farmers shortly after birth (53) or they are slaughtered at meat processing premises for human consumption or pet food, usually within the 1st week of life (26, 54). In New Zealand, ~2.2 million calves aged between four and ~7 days are slaughtered annually (26). These calves are at particular risk of welfare compromise, morbidity and mortality due to the very young age at which they are transported, mixed and held off feed prior to slaughter (14, 55, 56).

### On-Farm Calf Mortality

Livestock mortality rates are a useful, though somewhat crude, indicator of animal welfare on farm (57–59). Numerous observational studies document on-farm mortality rates in dairy calves and young stock [e.g., (60–62)] and causes of death are well-defined [e.g., (46, 60, 63)]. In general, calves are at greatest mortality risk during the first 4 weeks of life, with diarrhea and respiratory disease being the most important reasons for death (58, 64, 65). Risk factors for young cattle mortality are widely studied (60, 66–69) and include sociological factors such as farmer attitude (50) or “blindness” (70) toward animal welfare.

#### Management of Male Compared to Female Calves on Farm

There is evidence of discrimination against male compared to female dairy calves in several areas (6, 48, 67, 71). In particular colostrum and post-colostrum feeding practices differ between males and females in many countries [Canada: (72, 73); United States; (71); New Zealand: (48); Ireland: (69) and UK: (74)]. This can be associated with differential rates of failure of passive transfer (FPT) of immunologlobulins between male and female calves (75, 76). Although a recent Irish study found no difference in rates of FPT between male and female calves on dairy farms (68). In another study, which investigated health outcomes in surplus dairy calves at auction in Canada, there was a protective effect of being a female calf on the odds of omphalitis and being generally unhealthy (77). These authors also surmised that female calves were associated with a higher sale price because there was a perception that they received better care on farm.

#### Mortality Rates of Male vs. Female Calves On-Farm

It is likely that different mortality outcomes reflect differential treatment of male and female calves on farm (57). Notwithstanding the biologically higher risk of mortality in males, higher than expected mortality rates in male compared to female dairy calves are widely reported [(78) [at the receiving veal farm]; (61) [first month of life at mortality odds ratio of 1.20]; (79) [first 48 h of life]; (80) [UK; between 21 and 90 days]]. Additionally, Hyde et al. (80) reported that the trend for males to have a disproportionally greater rate of mortality increased from 17.4% in 2011 to 26.16% in 2018. In Ireland, Ring et al. (81) showed generally higher odds of male calf deaths compared to females in both dairy and beef herds. However, in dairy herds the odds of male calves dying compared to females was 6.15 compared to 3.34 in beef herds. Findings from both of these studies suggest that in accordance with others (82, 83) higher mortality rates in males from the dairy herd mirror risk factors associated with economic value as well as biology. Interestingly Ring et al. (81) also showed general higher risks of mortality of calves in herds with Jersey genetics. This would seem consistent with the very low economic value of both male and non-replacement female Jersey calves (84). Irrespective of sex, calves from herds with calf mortality problems are likely to have greater risk of morbidity given the causal continuum between morbidity and mortality. Thus, young calves presented for slaughter from farms with high calf mortality problems are more likely to have ante and PM indicators of poor health and welfare.

## Pre-Slaughter Mortality in Surplus, Unweaned Calves and Related Risk Factors

Pre-slaughter mortality includes calves that do not survive the journey to the processor or their time in the lairage yards prior to slaughter or that are so seriously compromised that they are condemned (euthanised) on arrival (26, 47). Both these authors reported that the latter category composed two thirds of all pre-slaughter mortality. Risk factors for increased pre-slaughter calf mortality may act on the farm of origin, in transit or upon arrival in the abattoir. There are very few studies investigating mortality and associated risk factors in surplus calves prior to slaughter. Data on such animals can yield important insights on calf welfare because of associations with standards of calf management on-farm, in transit and at the abattoir [e.g., (26, 48)]. Hence, pre-slaughter mortality, particularly when combined with the associated PM examination results of animals that died (85), is an important indicator of calf welfare. However, a calf can suffer poor welfare before slaughter without dying so it can be a crude indicator of animal welfare status. Hence, there is a need for validated ABI relevant to calf health and welfare to understand the experiences of calves that survive to the point of slaughter. Another constraint to the usefulness of pre-slaughter mortality is its comparative rarity in slaughter calves. An Australian study reported a pre-slaughter mortality rate for bobby calves as 0.64% (86) which was similar to that reported in a New Zealand study conducted in 2011 [0.7% (47)]. Following the introduction of legislation protecting bobby calf welfare in New Zealand, the national pre-slaughter mortality rate declined from 0.25% in 2015 to 0.06% in 2017 (24, 25). Given that slaughter calves are older in countries governed by EU legislation, the pre-slaughter mortality rate is likely even lower though there are no data readily available to support this theory. However, while the mortality rate is extremely low, this does not obviate the need to reduce it further. Understanding and addressing the underlying risk factors in each stage of the supply chain: on-farm, during transport and in lairage at the slaughterhouse can help to achieve this.

### Risks Associated With Management, Housing and Feeding of Calves on Farm

Calves slaughtered at 3–4 weeks of age are likely slightly better able to withstand the stresses of feed withdrawal, transport, movement through markets, lairage and slaughter than 3–4-day-old calves (87). However, the period on-farm when the welfare of these low value animals could be compromised is longer. Welfare concerns for dairy calves on farm are associated with housing, feeding and management practices (88). Apart from general calf management (e.g., nutrition, housing, etc.), management of ill-health can be a risk factor for poor calf welfare. This can be caused by mis-diagnosis and possibly incorrect therapy of a particular condition (e.g., sepsis) (89). But it can also mean that the problem was not recognized or that there was failure to treat (70). There are very few studies linking mortality of unweaned, surplus calves prior to slaughter with specific on-farm practices. The only on-farm risk factor identified by Boulton et al. (26) was time in the farm of origin's calving season which they suggest reflected farm-management related factors that change over the season. The severity of infectious disease in calves is influenced by management and hygiene practices (90) as well as immune status (91). Furthermore, disease transmission among infected calves may also be affected by management factors such as housing, group size and hygiene (92–94), which change over a farm's season (68, 70). Furthermore, research into the prevalence FPT of maternal antibodies in New Zealand dairy calves found that FPT was more prevalent in the middle compared to the early calving period (53). It is not routine practice to measure FPT on the vast majority of dairy farms internationally (70), hence farmers are not aware of the dynamics of this risk factor. The increase in FPT over the calving season observed by Cuttance et al. (53) may have contributed to the observed seasonal effect on risk of pre-slaughter mortality in the study of Boulton et al. (26).

### Risks Associated With Transport and Lairage

Not surprisingly, and in line with most other classes of animals, there is a correlation between increasing transport distance (from farm to processor) and the correlated travel duration and calf mortality (26, 86, 95, 96). Transportation of young animals from the farm to the processor imposes stressors that affect their biochemical, hormonal and metabolic status (97). Loading and unloading (86, 98), novel human-animal contact (99), and the inability to lie down (96) are major stressors with negative effects on calf health and welfare resulting in increased mortality. The slaughter schedule is the main risk to calf welfare associated with the slaughter facility itself (26). Given that calves in lairage yards don't have access to feed this risk is directly associated with the amount of time elapsed since milk withdrawal whereby longer lairage times are associated with longer time off feed. Prolonged feed withdrawal negatively impacts on calf energy status (100). Additionally, although there is access to water in lairage yards calves may not consume this such that water loss and dehydration are also associated with prolonged feed withdrawal (101). Clearly, in order to reduce slaughter calf mortality and morbidity, transport distance/duration should be as short as possible.

## Review of Ante and Post Mortem ABI Findings in Unweaned Slaughter Calves

### Ante-Mortem Findings

There are only two recent studies specifically concerned with ABI findings in calves at the abattoir (26, 47, 48). In relation to AM findings, Thomas and Jordaan (47) reported some observations on calves that died pre-slaughter and which were subjected to PM examination. Boulton et al. (26) reported the most frequently recorded ABI as correlates with calf mortality prior to slaughter as weakness, recumbency, emaciation and dehydration. These authors also included behavioral measures related to posture and oral behaviors in their lairage inspection of bobby calves (48). They reported that welfare-related conditions affected 20% of calves and concluded that more calves with compromised welfare were recorded than would be registered officially.

In New Zealand, calves must not be moved off farm younger than 4 days of age. However, neonatal characteristics in such animals are commonly reported findings. For example, Thomas and Jordaan (47) observed “wet” umbilical cords in 25% of calves that died pre-slaughter. Similarly, Stafford et al. (102) classified over 4% of calves as “marginal” because of at least one of the following: wet umbilicus, hollow-sided, apparently immature, or weak and slow and unsteady on their feet. Studies conducted on unweaned calves presented for auction to the veal industry also report neonatal characteristics (12.3% of calves inspected) on the basis of wetness of the umbilical cord (77). At birth the umbilical cord is wet and though of variable length and diameter will be, on average, 15–25 mm thick (diameter) close to the base, (103). With age the cord dries [on average, by day 3, in all, by day 7; (104)] and shrivels from the distal end thus reducing diameter, e.g., 5–10 mm at 24–72 h old (83) and 10–15, 5–10 and 5–10 mm at one, 2 and 3 weeks of age (105). Cord dryness alone is a poor indicator of calf age (104). The cord finally detaches, on average, at 15–20 days old (103).

Findings from studies of young calves at veal auctions can help inform likely AM findings in unweaned slaughter calves. Both Marquou et al. (77) and Wilson et al. (106) report navel infection or omphalitis caused by opportunistic bacteria (107), as the main finding in such animals. Both studies also reported concerns with lightweight calves as these, and animals with navel infections, have reduced growth (108) and increased mortality (67) at veal farms.

### Post-mortem ABI Findings in Unweaned Slaughter Calves

The value of necropsy findings from calves that die on farm in informing health management plans is well-recognized [e.g., (109)]. In slaughter animals, PM checks are primarily motivated by food safety (30) but meat inspection also provides an excellent opportunity to measure ABI of relevance to calf welfare [e.g., (44, 45)].

The most frequently recorded PM findings in calves that die (or are euthanised) pre-slaughter are digestive tract disorders and inflamed/infected umbilicus (omphalitis) (26, 47). Only Thomas and Jordaan (47) report recent findings from routine PM examinations of surplus calves. They found that omphalitis (54%) and septicaemia (37%) were the main causes of calf condemnation post-slaughter. This is in line with older studies performed in New Zealand (110, 111). However, Thomas and Jordaan (47) recorded proportionally more omphalitis and less pneumonia than these studies. They discuss that the younger age of calves in their study was likely responsible.

Omphalitis represents a major difference between PM findings of bobby calves and other classes/ages of calves. As mentioned above it is likely that the young age of these calves at slaughter is responsible such that infection has not yet traveled beyond the umbilicus and become systemic. In older calves, infections that originated in the umbilicus could be responsible for the systemic infections reported [i.e., septicaemia; (83, 112) or idiopathic peritonitis; (113)]. Additionally antibiotic usage is uncommon in bobby calves (48) compared to in calves destined for intensive beef rearing or veal systems (114–116) which could also explain the omphalitis-related pre-slaughter mortality and post-slaughter condemnations.

Thomas and Jordaan (47) describe how the few cases of pneumonia they recorded were considered typical of those caused by the aspiration of food material, probably during esophageal feeding, rather than the enzootic-form typically associated with calf pneumonia. The low incidence of enzootic pneumonia in bobby calves is in line with their young age (63, 117, 118). Pneumonia occurs at a much higher prevalence in all classes of older calves [dairy replacement heifers: (60, 63, 64), beef calves: (10, 118), veal calves: (83, 113, 114)].

In the absence of data on PM findings for older unweaned slaughter calves (i.e., in the EU/Irish context), we rely on data from two recent sources. O'Donovan (117) reported the main causes of on-farm mortality in calves, in various age categories, submitted to the six Irish government veterinary laboratories for diagnosis of the cause/s of death. While Mee (70) reported morbidities recorded in dairy calves from 3 days to 3 months of age on 120 Irish dairy farms (Table 1). The majority of deaths were due to infectious causes (Table 2). In line with findings for bobby calves (47) the main finding in calves 0–1 month of age was infection of the gastrointestinal tract. However, respiratory tract infections, though less common in calves in the 0–1 month age group compared to the 1–5 month age group (Table 2), were a more common cause of death than reported in the New Zealand studies. Though only 7% of calves were diagnosed with navel or joint ill, as discussed above, it cannot be discounted that the navel was the original point of infection in calves diagnosed with systemic infection (19.4%). The high proportion of farms with calves having navel ill further supports this theory [Table 1, (70)].

**Table 1:** Morbidities recorded in dairy calves from 3 days to 3 months of age on 120 Irish dairy farms (*n* = 6,850) (70).

**Level**	**%**	**Diarrhea**	**Respiratory disease**	**Navel ill**
Herd	Min.	0	0	0
	Max.	12	27	6
	≥1 calf	89	42	53
Calf	%	7	2	2

**Table 2:** Conditions most frequently diagnosed on *post-mortem* examinations of calves (0–5 months old) which died on Irish farms, (*n* = 1,219) (117).

**Five most common conditions**	**0–1 month *n* = 609 (%)**	**Five most common conditions**	**1–5 months *n* = 610 (%)**
Gastrointestinal infection	27.2	Respiratory infection	30.6
Systemic infection	19.4	Gastrointestinal infection	13.4
Respiratory infection	11.0	Gastrointestinal torsion/obstruction	9.4
Navel/joint ill	7.1	Systemic infection	9.1
Gastrointestinal torsion/ obstruction	6.9	Gastrointestinal ulcer/perforation/foreign body	6.1

Hence, these data provide a good indicator of likely PM findings in slightly older though still unweaned, slaughter calves. On the basis of the findings outlined above we propose a range of ABI for potential recording at AM and PM examination in unweaned calves in the following sections.

## Abattoir-Based ABI Relevant to Calf Health and Welfare

### Ante Mortem

#### Group Based Measurements

Inspection of calves at unloading could be on a batch-basis to identify problem cohorts for more detailed inspection. However, there are a number of behavioral ABI to measure at unloading which reflect not only fearfulness but also the efficiency and care with which calves are handled on arrival at the slaughterhouse (119, 120). Indeed slaughter plants are rarely designed with the behavioral needs of animals in mind (120). For young calves, unloading could be even more stressful than the journey itself (121). Ideally group-based behavioral indicators would be employed at this point such as number of falls, slips, jumps, balks, reversing, mounting and vocalizations (99, 121, 122). Unloading is a particularly useful opportunity to identify severely compromised calves such as those appearing very unsteady/lame, and falling frequently. Group-based behavioral observations can also be conducted in the lairage pens (48). In that report, observers viewed groups of calves from outside the pen using binoculars. They employed a detailed list of behavioral ABIs including group–(huddling and social play) and individually–based behaviors (oral behaviors, locomotory play, postures, head shaking or tilting). These measures could be useful on an *ad hoc* basis for specific welfare schemes/welfare assessments but would be logistically difficult for abattoir vets to conduct.

Human-animal relationship (HAR) tests measure calf fearfulness and reflect the way in which the calves were handled on farm (99) and in the abattoir (122, 123)]. Though measured on an individual calf basis (99, 124, 125) they could be conducted in the lairage pens and thus are considered as group (or pen) based observations.

#### Individual/Calf Based Measurements

The AM inspection is essentially a clinical examination of individual calves, preferably conducted by the lairage vet. As with all such examinations a systematic approach (where the same ABIs are evaluated in the same way in every calf) will glean a comprehensive picture of the animal's health and welfare status. It is also possible to conduct a systemic “walk-through” of the pen when carrying out the AM inspection of calves (48). Given the circumstances in which such an evaluation is conducted (large numbers of calves, limited space, time pressures), this is primarily a visual examination but with auxiliary examinations as indicated from the visual exam, e.g., palpation, temperature checking. Critical to the process is adequate lighting at the unloading dock and in the lairage. Each of the ABIs individually, but also collectively, inform judgement on whether, and to what degree, the calf's health and welfare is compromised. Thus, either an overall score could be assigned to each calf (e.g., normal/healthy or abnormal/unhealthy/condemned and euthanised) or only to those calves where poor welfare is recorded. Marquou et al. (77) assigned calves presented at auction for sale into veal production a general health score based on the summation of abnormal findings.

Hereunder we outline the main ABI to record in unweaned calves prior to slaughter with a brief explanation of their relevance to the overall health and welfare status of the calf. Examples of animal-based-indicators, their key features and published studies that used these indicators in a scoring system are shown in Table 3.

- Age/maturity (appearance of neonatal characteristics)

**Table 3:** Examples of animal-based-indicators, their key features and published studies that used these indicators in a scoring system.

**Animal-based indicator (ABI)**	**Key features of ABI**	**Example of study using this ABI in a scoring system**
Age/maturity	Umbilical cord characteristics	Hides and Hannah (104)
Demeanour	Inquisitiveness, responsiveness, posture, suck reflex	Barry et al., (68)
Body condition	Subcutaneous adipose reserves, sunken/hollow flanks	Renaud et al., (67)
Stability	Unassisted standing/tremor	Barry et al., (68)
Shivering	Shaking slightly and uncontrollably	Bellows and Lammoglia (126)
Injuries	Skin lesions	Jorgensen et al., (127)
Locomotory ability	Lameness, joint swelling, contracted tendons	Renaud et al., (67)
Cleanliness	Faecal soiling of the hair coat	Barry et al., (68)
Dehydration	Enophthalmos, skin tenting	Renaud et al., (67)
Nasal/ocular discharge	Excess/abnormal discharge	Renaud et al., (67)
Respiration	Breathing characteristics	Ministry for Primary Industries (48)
Umbilical abnormalities	Umbilical heat/pain/swelling	Renaud et al., (67)
Body temperature	Rectal temperature	Mahendran et al., (128)

Clearly determining calf age is important from the point of view of compliance with codes of practice or legislation governing minimal ages at which calves can be moved off farm. While it is not possible to be precise about a calf's age, (even from birth certificates as calves may not be registered for days after birth) certain indicators can be used to estimate post-natal maturity. Very young calves may still be wet and can have difficulty standing (77) but establishing wetness of the umbilical cord is the main indicator of maturity. As a heuristic, calves with a wet cord are less than a week of age, those with a dry cord are likely to be more than 3 days old and those without a cord are likely to be more than 2 weeks old.

- General demeanor and posture

In the absence of obvious clinical anomalies, a calf's general demeanor can indicate the presence of an underlying illness, stress or pain. While standing, a calf showing good demeanor is alert/bright, interested in its surroundings and inquisitive. It should show a good suck reflex and be responsive (i.e., moves away or toward) to the approach of a human. Stafford et al. (102) described such calves as “strong, walking freely, round-sided, bright and alert.” Such calves lie in sternal recumbence with their head held upright. In contrast, a calf with poor demeanor is dull and depressed, tilts its head downwards with drooped ears, and shows no interest in its surroundings, people or other calves. While lying such calves may tuck their head back on their shoulder or if in extreme pain, distress or illness will lie in lateral recumbence. They are either reluctant or unable to rise. A calf with abdominal pain or a thoracic disorder (e.g., pneumonia) may have a crouched posture with a humped back (kyphosis) while standing.

- Body condition

The body condition score (BCS) is an assessment of subcutaneous adipose reserves and therefore how well the calf was fed on-farm or the degree to which it has catabolised its fat reserves. This is best assessed by palpation of certain sites such as over the ribs, lumbar spinal processes and tail head. In a thin or emaciated calf which has catabolised its reserves there is less subcutaneous fat; a low BCS. Visual or palpation examination of the calf's abdomen to detect presence/absence of colostrum or milk/milk replacer in the gastrointestinal tract (sunken/hollow vs. full/rounded flanks) may support the findings of the BCS evaluation.

- Stability while standing

A healthy calf will maintain a standing position without obvious effort. A weak calf (e.g., due to under-feeding or diarrhea) or one with pain (e.g., due to a fracture) or hypothermia or with CNS abnormalities (e.g., cerebellar hypoplasia) may quiver/tremor while standing or shift legs uneasily.

- Shivering

If a calf is shivering (shaking slightly and uncontrollably) this suggests a degree of hypothermia (cold), which can be interpreted given the ambient conditions. Wet, small calves are at greater risk of hypothermia so coat condition also needs to be taken into account.

- Injuries and skin lesions

**Figure 1:**
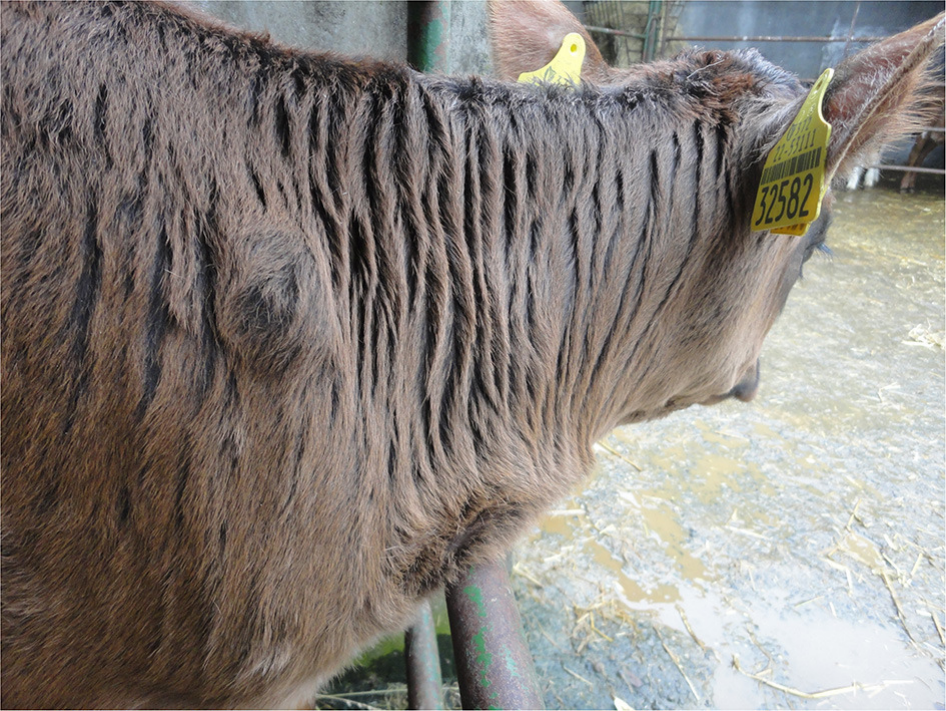
Injection site swelling under the skin on the shoulder.

**Figure 2:**
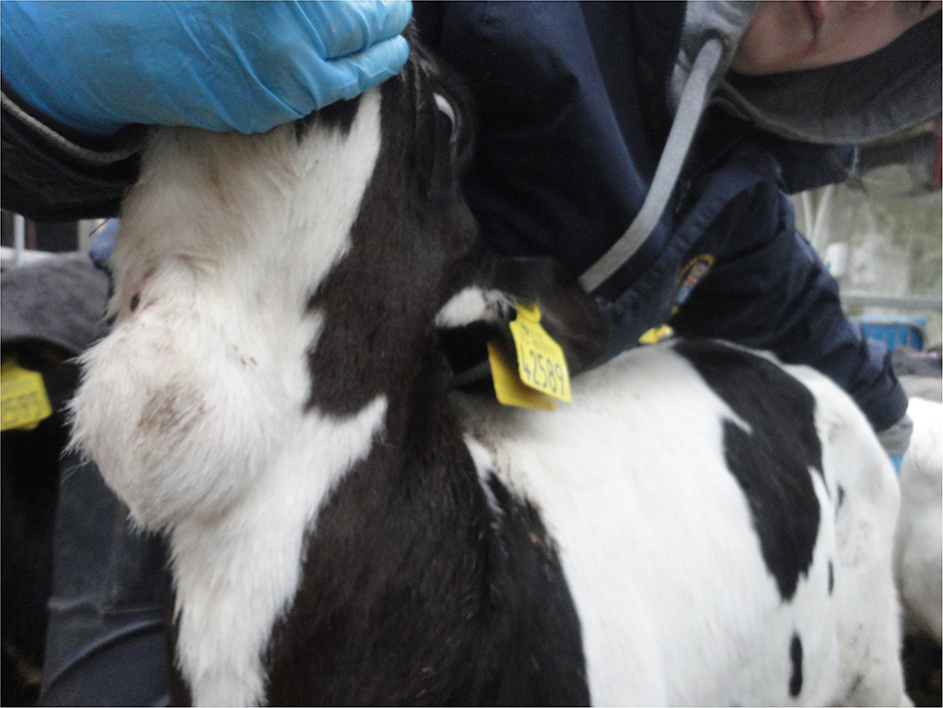
Abscess under the skin in the neck of a young calf.

When visually inspecting a calf, injuries may sometimes be apparent such as swellings (e.g., over joints in the case of joint-ill or subcutaneous abscesses or haematoma possibly following injection or tagging), (Figure 1) wounds/abscesses (e.g., from sharp surfaces or handling), (Figure 2) or hairless patches (focal alopecia, e.g., on the perineum from prolonged scouring). The extent and degree of such lesions may relate to other findings in the calf. Boulton and colleagues (48) employed a scoring system for skin lesions for calves adapted from one devised by Jorgensen and colleagues (127) for horses.

- Locomotory ability/joint swelling

A healthy calf will move freely while a calf in pain or with limb joint infection (joint-ill) or limb abnormalities (e.g., contracted tendons) will limp exhibiting varying degrees of lameness or limit its voluntary movement.

- Cleanliness/fecal soiling

**Figure 3:**
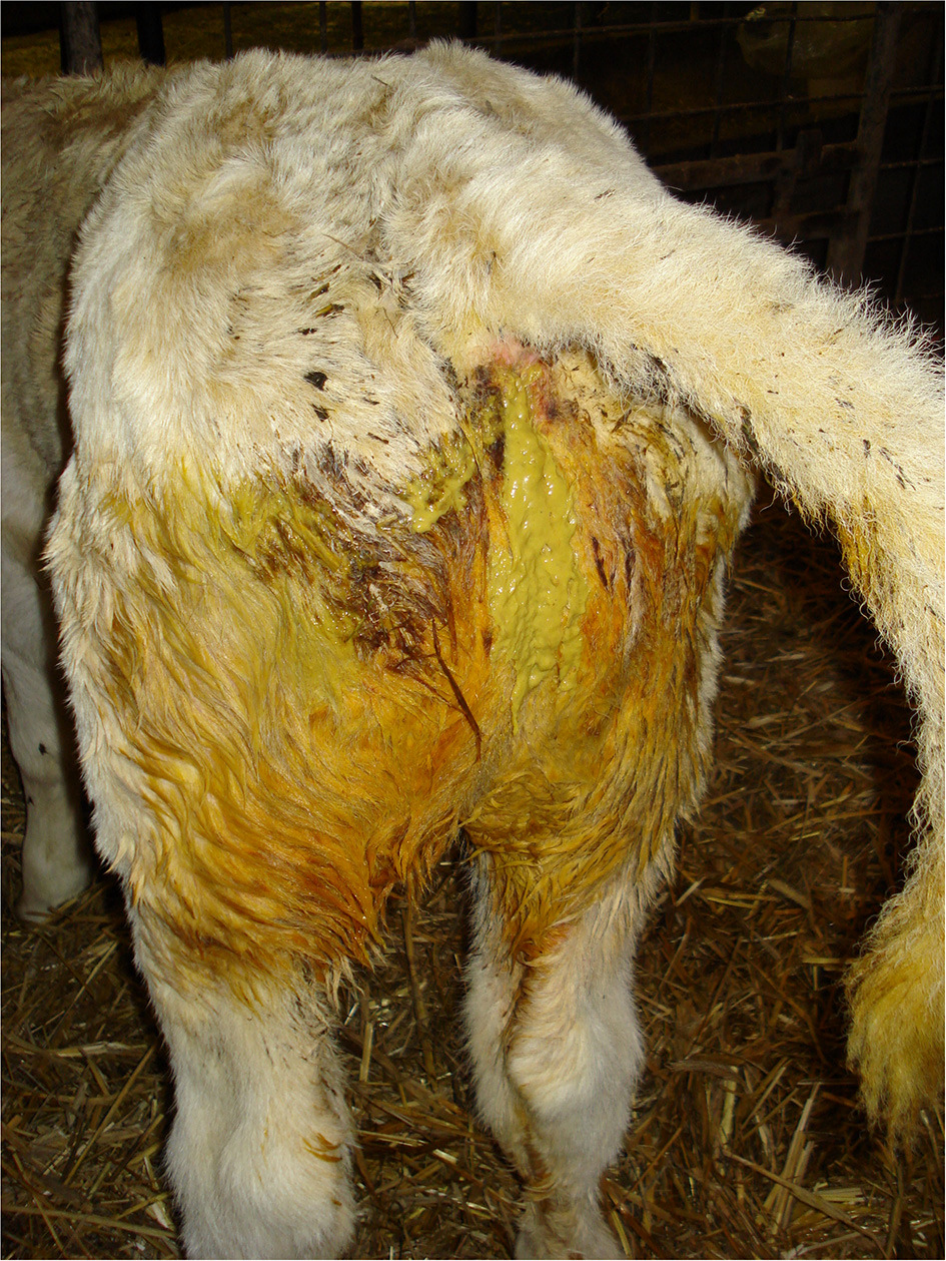
Perineal fecal soiling in a calf with diarrhea.

Depending on the type and cleanliness of the on-farm bedding or transport vehicle, calves should have a clean coat. Perineal soiling with watery or bloody feces indicates diarrhea (scour), (Figure 3) and perineal alopecia indicates chronic diarrhea. Visual observation of fecal consistency accurately correlates with reduced fecal dry matter content and diarrhea (73). Thomas and Jordaan (47) reported that the majority (96%) of calves condemned due to digestive tract disorders (recorded PM) presented with severe diarrhea AM.

- Dehydration

**Figure 4:**
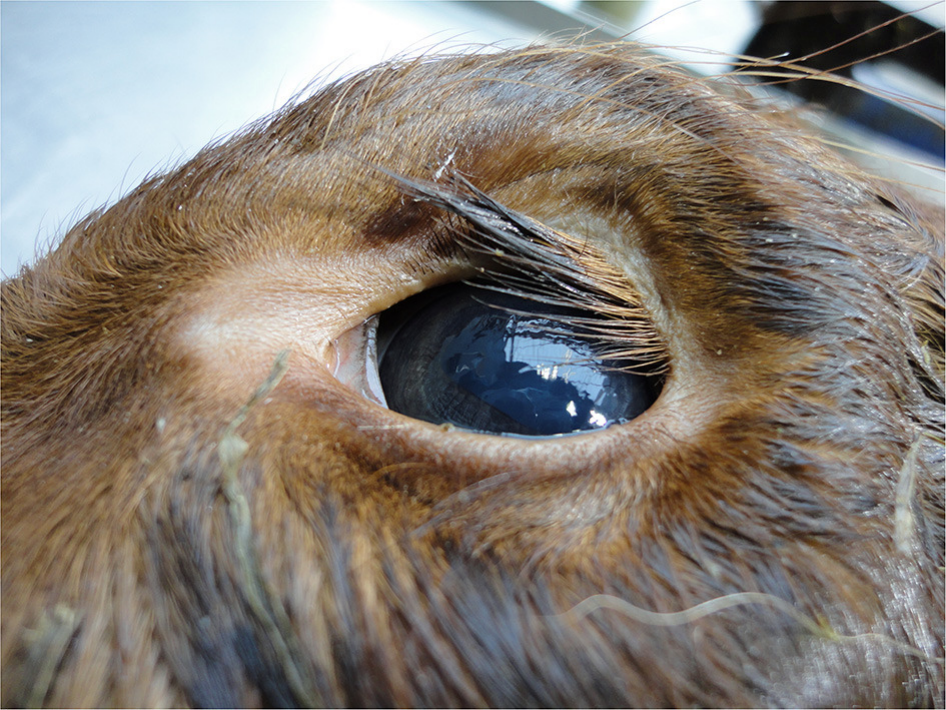
Enophthalmos (sunken eye) in a calf with dehydration.

The hydration status of a young calf should not be obvious unless the calf is dehydrated, as the calf will appear normal. However, dehydration is not uncommon in calves transported for long distances with an inadequate water supply. Dehydration (commonly due to diarrhea but also peritonitis and prolonged inadequate fluid intake) can be diagnosed visually by the degree of enophthalmos (recession of the eyeball into the eye socket), (Figure 4) skin tent test and capillary refill time (101). With the skin tent test the skin over the thorax is raised and the return time measured; <2 s indicates the calf is <5% dehydrated (normal) while >5 s indicates a calf is >10% dehydrated (obvious dehydration), (67).

- Nasal, ocular discharge

**Figure 5:**
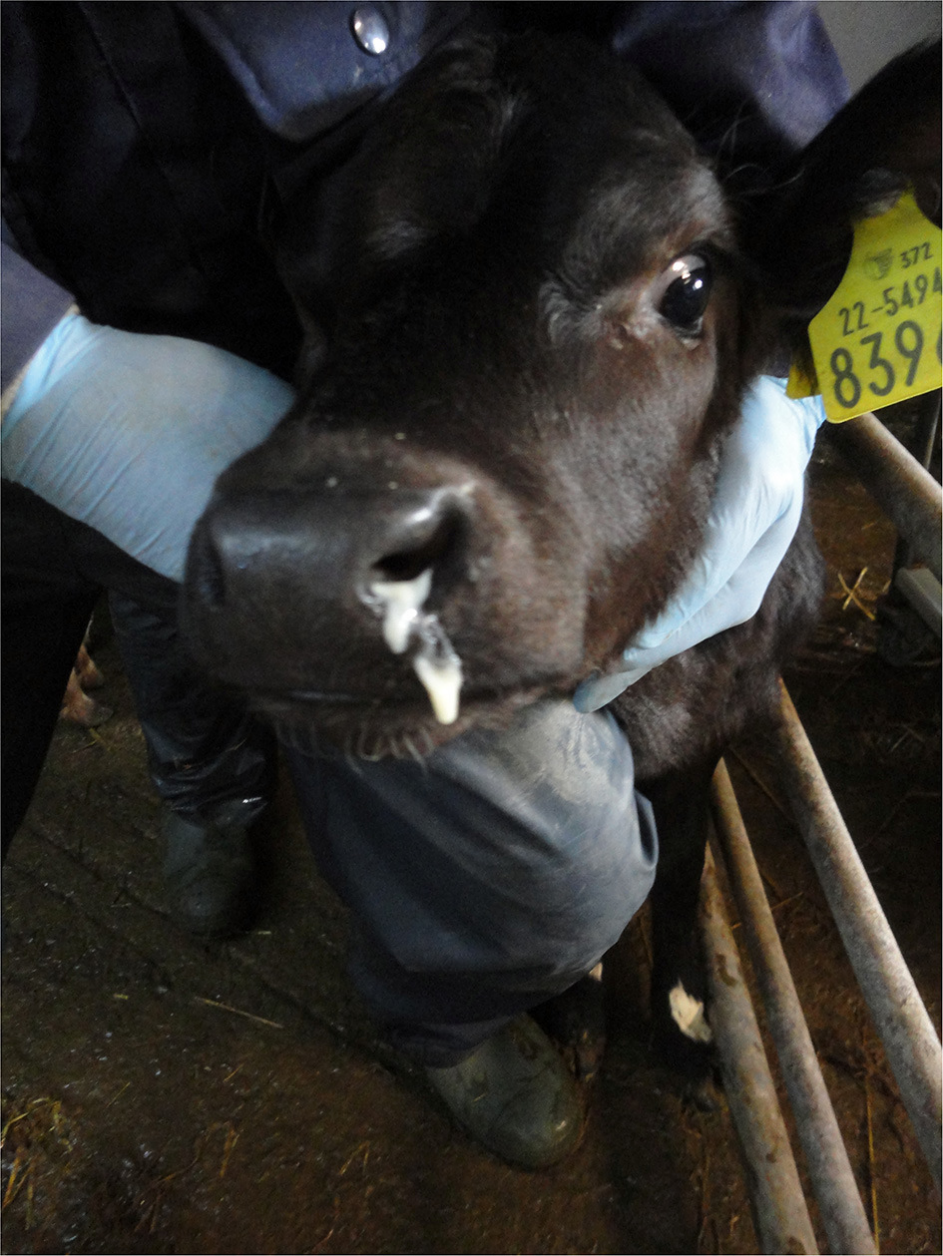
Nasal discharge in a calf with respiratory tract infection.

**Figure 6:**
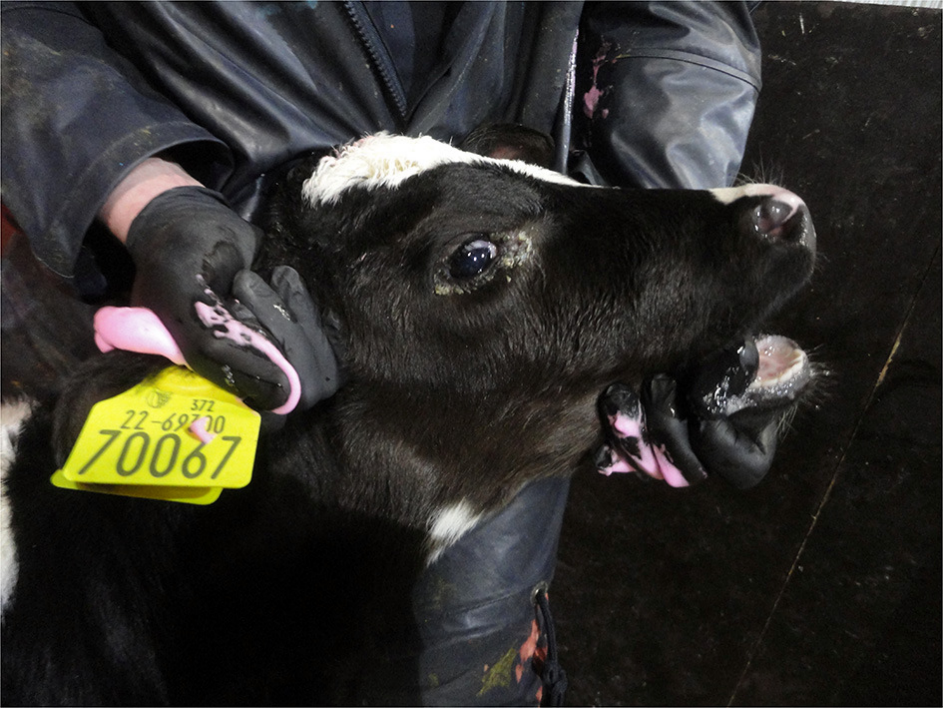
Ocular discharge in a calf.

The presence of a nasal (Figure 5) and/or ocular discharge (Figure 6) (usually bilateral) indicates upper respiratory tract infection. Respiratory tract infections reflect both the infectious challenge from the calf's environment (e.g., poorly ventilated housing) and the livestock in a common air space (especially where there is overcrowding or older stock are present). The nature (e.g., nasal discharge–serous, cloudy, mucopurulent, purulent) and extent of the discharge may indicate the severity and chronicity of such infection. Rarely, nasal discharge with milk may indicate palatoschisis.

- Respiration

**Figure 7:**
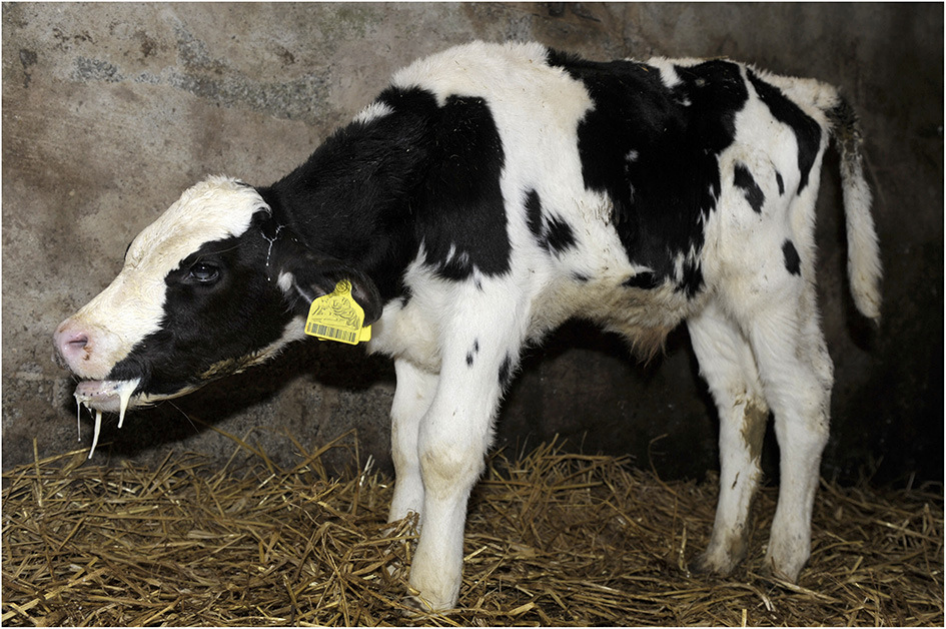
Coughing calf with respiratory disease (Animal Health Ireland).

While the normal respiratory rate is approximately 10–30 bpm (129), this is affected by numerous factors such as recent exercise, transport, ambient temperature and time of day. Pathological factors which can elevate respiratory rate include lower respiratory tract disease and pain. Calves should not normally pant [>36 breathes per minute, counted over a 20 s period as per (48)] or spontaneously repeatedly cough (Figure 7) so the occurrence of either indicates respiratory compromise.

- Umbilical abnormalities

**Figure 8:**
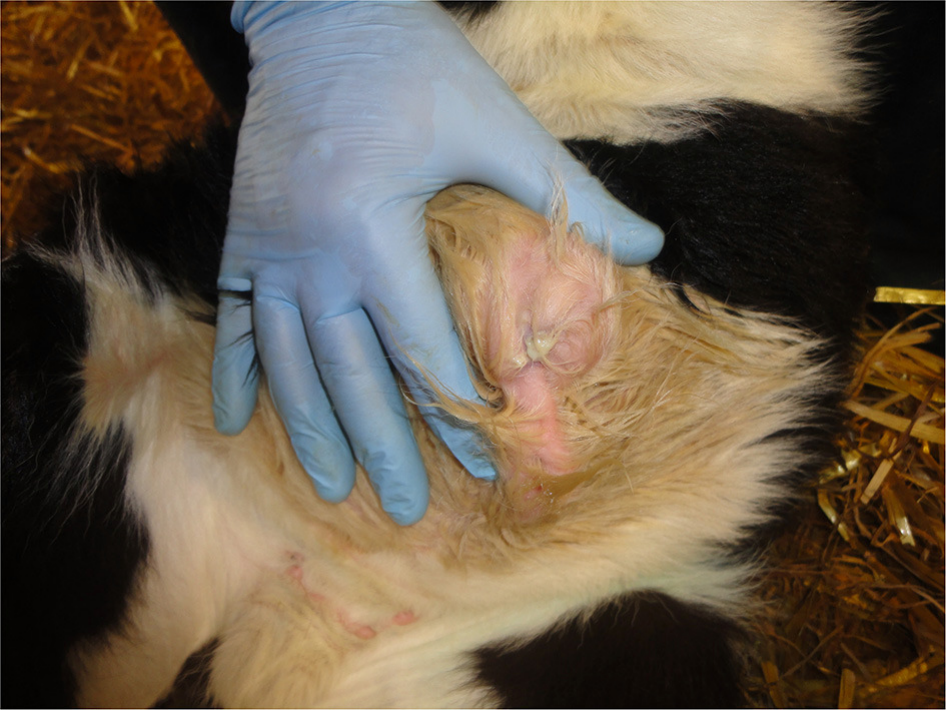
Infection of the umbilicus (navel ill).

Common abnormalities of the umbilicus such as infection (navel-ill/omphalitis) and/or umbilical herniation are best detected by palpation (Figure 8) rather than just relying on observation.

- Rectal temperature

In calves where visual inspection suggests an infectious process (e.g., navel-ill, joint-ill, pneumonia, diarrhea, etc.), (or possibly hypothermia), measurement of rectal temperature is warranted. In the normal young calf this will be <38.5°C (128) but, recent transport and exercise may elevate normal rectal temperature.

Following this systematic examination it is possible to establish if the calf is healthy, its approximate age, whether it has been adequately fed, and whether it is suffering from injuries, infections or congenital defects. Thus, each calf can be scored on its health and welfare status prior to slaughter.

### Post-mortem

As with the AM inspection, the PM inspection is at the animal level. PM indicators may confirm findings from the AM indicators or add additional information about the calf's nutritional, infectious, injurious or developmental status not detectable from the AM evaluation.

Unlike a necropsy, where a more forensic approach is taken to investigate the carcass, abattoir carcass inspection is subject to the limitations of the conditions under which it is conducted. These include limited inspection time per carcass, the skin and musculoskeletal system separated from the viscera, a moving carcass or viscera line and no control over carcass opening and inability to collect confirmatory samples. Additionally any artifactual changes introduced by the method of killing and hanging the carcass need to be considered when evaluating the carcass for abnormalities.

Salient ABI detectable at PM calf inspection, their links to farm management and relevance for calf welfare are outlined hereunder.

- Umbilical disorders

**Figure 9:**
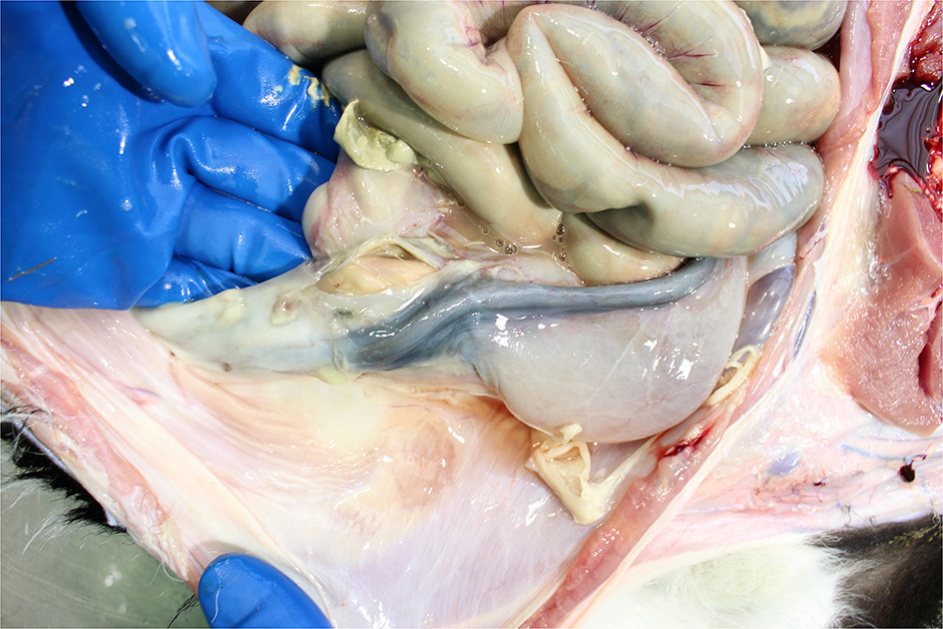
Infection in and around the umbilicus (omphalitis).

Omphalitis is an infection of the umbilicus (Figure 9) that may be localized to the umbilicus or track up along the umbilical arteries to the bladder and pelvis or along the umbilical vein to the liver causing secondary site infectious foci. This has serious welfare implications for the affected calf due to chronic pain and resultant ill thrift. There are numerous on-farm risk factors for such infections, including umbilical antisepsis, colostrum management and feeding practices (and associated passive transfer) and general hygiene practices (93, 94).

- Lung disorders

**Figure 10:**
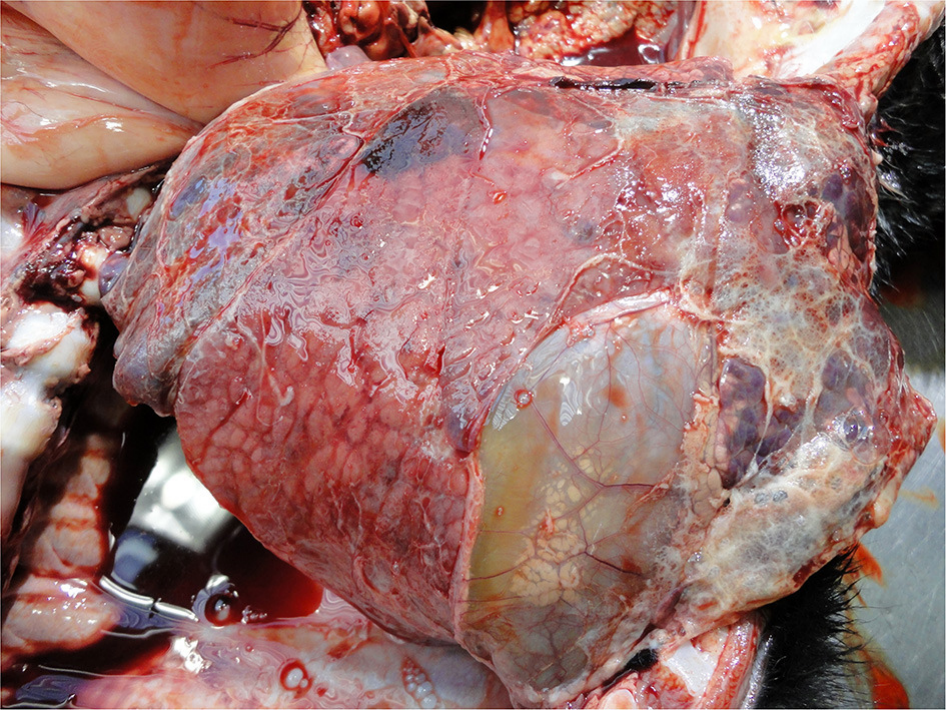
Pneumonia and pleurisy in a calf with respiratory infection.

Pneumonia and pleurisy are the most common visible lesions in calves with lung disorders (Figure 10). These reflect inadequate diagnosis and/or therapy of respiratory disease, usually on a group basis as well as a myriad of on-farm calf management and housing practices (44, 83, 130). Where pulmonary lesions are detected at PM inspection it is likely there are other sub-clinically affected calves in the same environment also with compromised welfare.

- Abomasal contents and disorders

**Figure 11:**
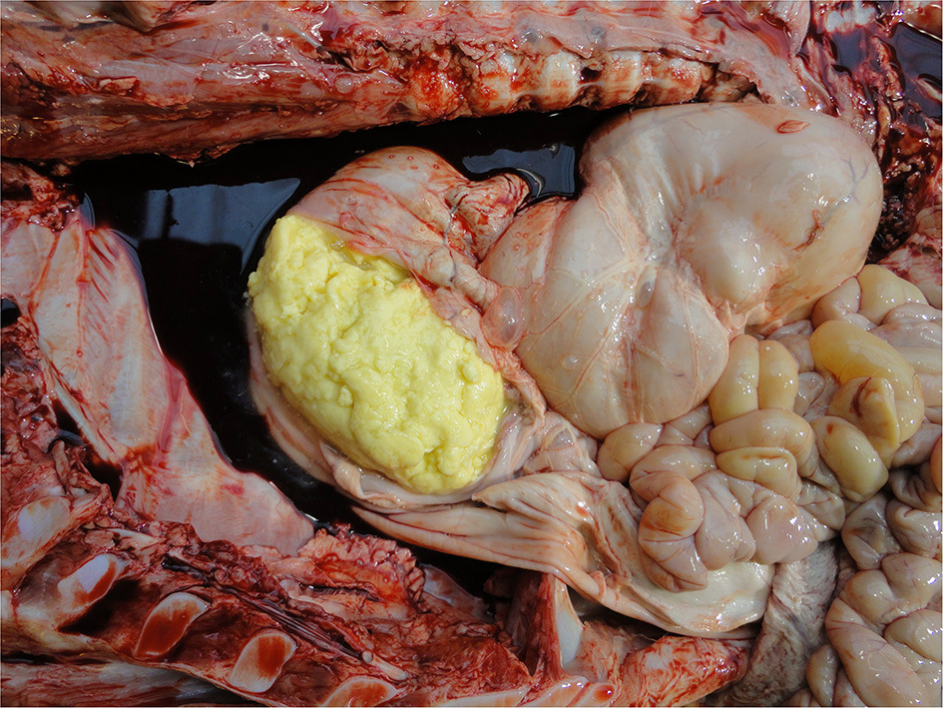
Well-formed curd in the abomasum of a young calf.

Incision of the abomasum (subject to abattoir meat inspection SOPs) reveals how recently the calf was fed and what it was fed. Normally the abomasum should contain variably formed rennin curd and whey (Figure 11) though there may be evidence of oral electrolyte administration (depending on the color of the electrolyte product). The latter reflects recent therapy for dehydration, e.g., calf diarrhea. Given that the median time for complete abomasal emptying is 1.5 days (131), an empty abomasum suggests the calf was not fed recently. While absence of abomasal curd may rarely reflect abomasal dysfunction resulting in failure of curd to form (132) or the use of non-clotting (usually whey-based) milk replacers (133), whey would still be present if the calf was fed recently. Post-mortem examination alone cannot distinguish between these underlying causes, but the absence of curd prompts questions about the feeding practices used on-farm. Inspection of the abomasum can also reveal mucosal pathologies (oedema, hemorrhages, and ulceration of varying degrees including penetrative with localized or generalized peritonitis). Bedding material may be found in the abomasum from about a week of age and occasionally hair balls (tricholiths), abomasal bloat, or torsion may be found in older calves. Abomasal disorders in young calves are a reflection of both suboptimal feeding management (particularly with automatic milk/milk replacer feeders) and poor hygiene of the calf's feeding environment (134).

- Intestinal contents and disorders

Enteritis is the most common lesion found in the calf's intestines though congenital defects (e.g., intestinal or anal atresia or stenosis) are found occasionally. Enteritis is visible as fluid-filled contents with variable congestion of the intestinal serosa and mucosa and enlargement of the intestinal lymph nodes. Thomas and Jordaan (47) reported that the majority (96%) of calves that died pre-slaughter and were diagnosed with digestive tract disorders (usually without macroscopic enteritis) PM presented with severe diarrhea on arrival at the slaughterhouse. Calf diarrhea (enteritis) is caused by infections (e.g., cryptosporidia, coccidia, rotavirus, etc.) the calf picks up from its environment, and its inability to protect itself against these common agents (i.e., its immune status). Thus, the presence of enteritis reflects both inadequate colostrum management and/or an excessive infectious challenge in the calf's environment. Enteritis is a painful, debilitating condition causing ill thrift and seriously compromising calf welfare.

- Fat reserves

Fat reserves can be assessed from the perirenal, epicardial, mesenteric, intrapelvic (brown fat—required for non-shivering thermogenesis) and subcutaneous (white fat) deposits. In cases of catabolism, reserves may be visibly depleted from about 2 weeks of age indicating either under-feeding and/or a debilitating process, e.g., infection. Fat color varies with breed, e.g., more yellow in Jerseys (135).

- Rumen contents and disorders

In younger calves, the presence of milk in the rumen is cause for concern (47). Milk in the rumen of calves <1 month old reflects failure of the esophageal groove to close properly and to deliver milk directly into the abomasum. Some calves have a poorly functioning esophageal groove (136), potentially explaining this finding, but feeding of the calves via an esophageal feeder [as described by Chapman et al. (137)] prior to transport to the abattoir may also cause this to occur. In some calves (“rumen drinkers”) an excessive, acidic/sweet smelling, milk volume is present in the rumen. This is found in calves which are repeatedly and/or over-fed using an oro-gastric feeder (“stomach tube”) such as when they fail to suck adequately.

- Peritonitis

**Figure 12:**
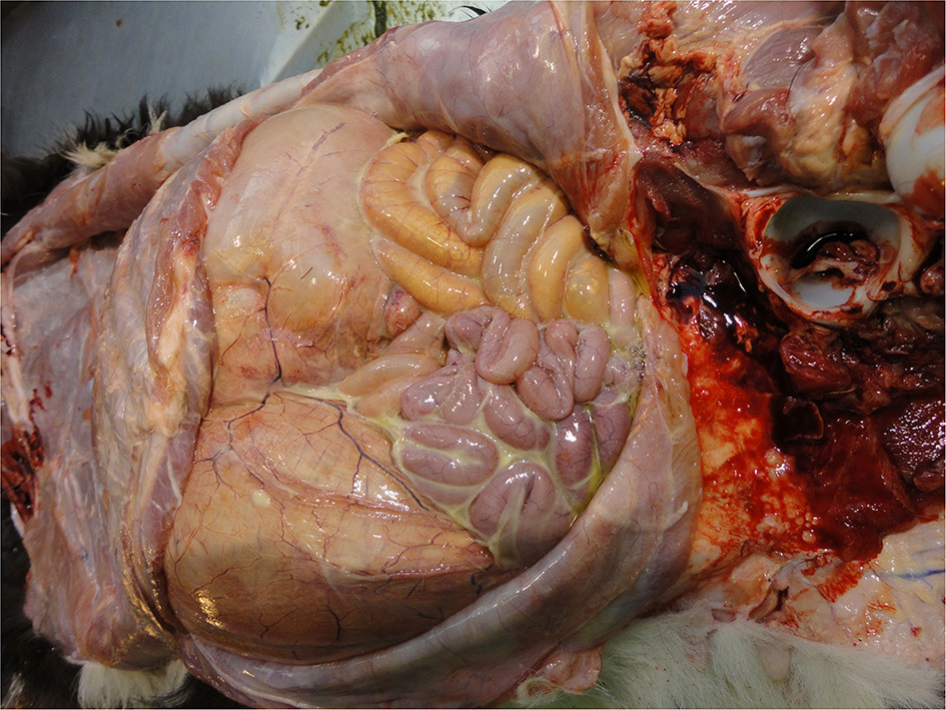
Purulent peritonitis in the abdomen of a calf.

Infection of the abdominal peritoneum is usually secondary to a primary infectious focus elsewhere, e.g., umbilicus or liver or it may be part of a generalized infection, e.g., sepsis. It may be localized or extensive depending on the chronicity and severity of the infection and can vary from serous to fibrinous to purulent (Figure 12). Detection of such severe pathology at the PM inspection reflects inadequate management of diagnosis and therapy. Peritonitis is a painful condition indicating severe welfare compromise.

- Septicaemia

**Figure 13:**
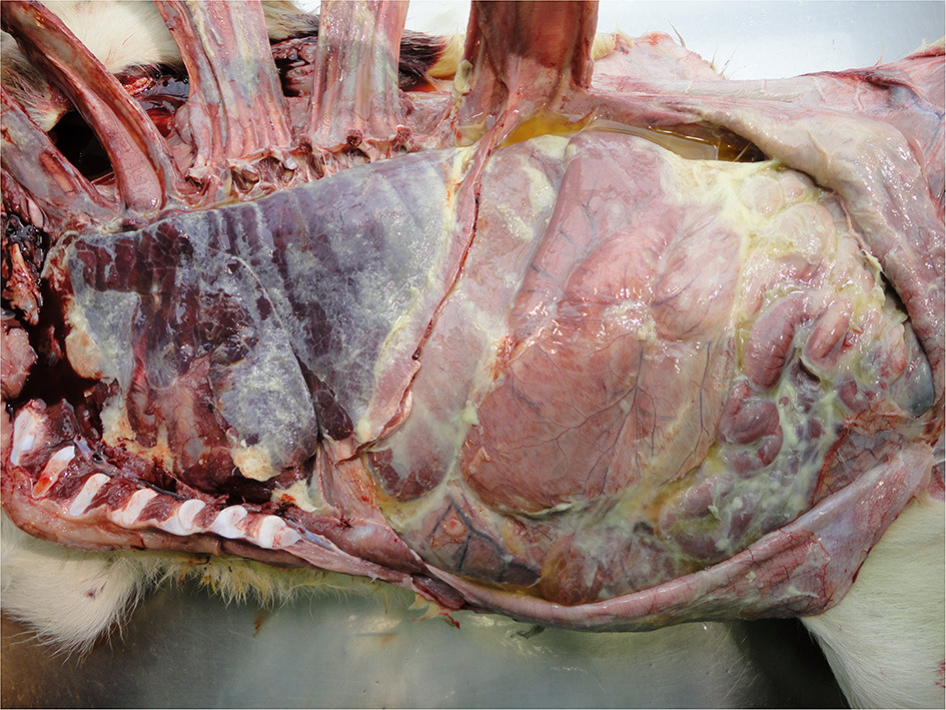
Sepsis affecting the thorax and abdomen of a calf.

Infection that has spread to multiple organs (sepsis) may be detectable from the congested appearance of these organs (e.g., lungs, liver, intestines, spleen, kidneys), and associated lymph nodes and the presence of fibrin deposits (Figure 13). The presence of sepsis at PM inspection reflects overwhelming infectious challenge from the calf's environment and/or compromised immune status and severely compromised welfare.

- Arthritis

**Figure 14:**
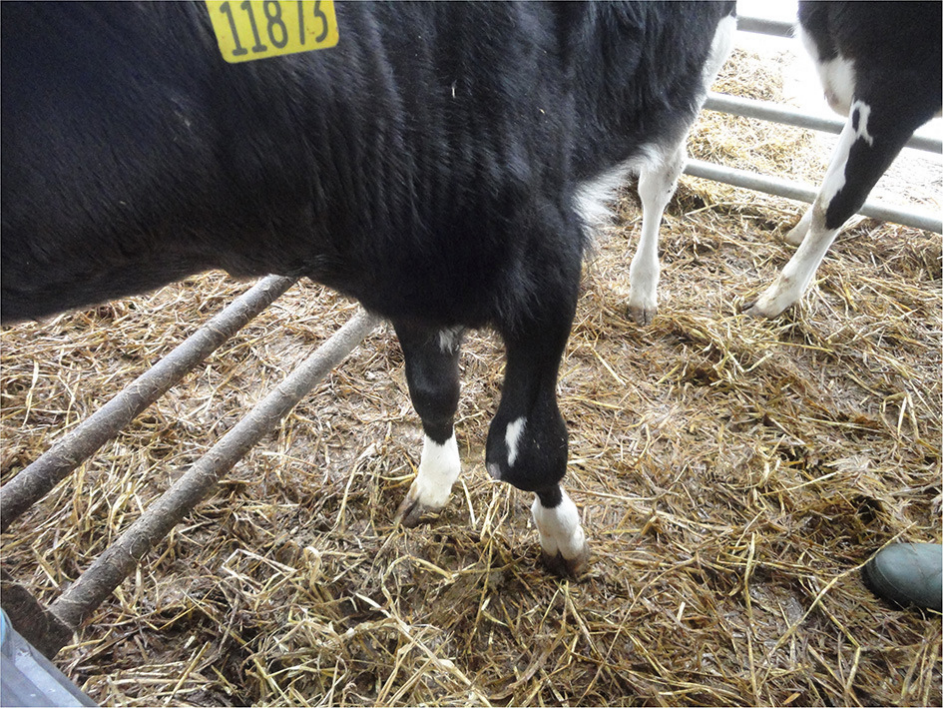
Joint ill causing swelling of the carpus in a young calf.

**Figure 15:**
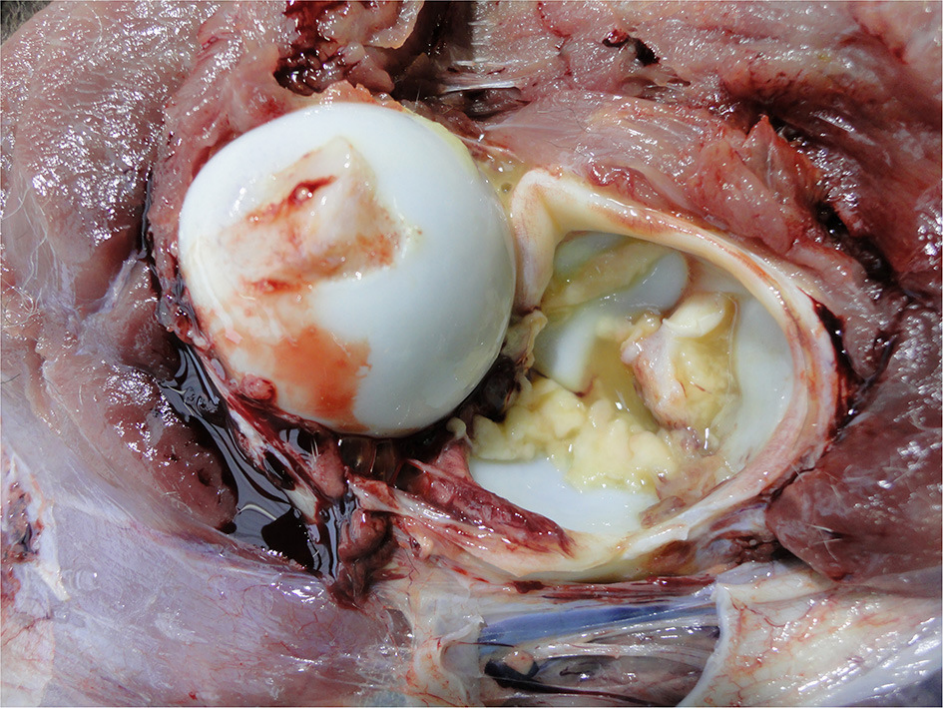
Infection in the hip joint (arthritis) in a calf.

As arthritis is an extremely painful condition (138) it reduces calf welfare. It most commonly reflects poor environmental hygiene and/or poor perinatal umbilical/colostral management and consequent joint infection by opportunistic environmental pathogens. It can also be caused by the presence of specific primary pathogens, e.g., Mycoplasma bovis, in the herd (139) which may be transferred to calves through colostrum, waste milk or environmental contamination. Relying on detection of lameness and joint swelling in the live animal underestimates the presence of arthritis in young calves (47). However, arthritis cannot be detected PM unless joints are routinely incised, except in cases with obvious joint swelling, discharge or other signs of infection. While these signs may be obvious in lower limb joints (Figure 14) they may be more difficult to detect in the upper limb (Figure 15) and spinal joints. Thomas and Jordaan (47) found that arthritis most commonly affected the tarsal joints.

- Fractures

Fractures are rare in young calves but can occur following traumotocia or postparturient accidents in the ribs, limbs or mandible. They may occur on-farm or during transport where they may reflect unsuitable transport conditions or mis-handling. They obviously seriously impair calf welfare.

- Abscesses

Foci of infection (abscesses) may occur in any organ internally or externally. Externally, they are likely to result from trauma, poor injection or tagging technique or umbilical infections. Internally, they may result from systemic infections or localized infections, e.g., in the liver or lungs or in the neck from esophageal rupture following faulty oro-esophageal feeding technique. While internal abscessation is not possible to diagnose specifically, affected calves may show signs of non-specific ill-thrift with poor body condition. Where multiple calves in a batch have abscesses at the same site, e.g., injection site in the neck, this indicates poor technique with resultant localized pain and reduced welfare.

## Calf AM/PM Welfare Scheme

### Validation of ABIs

None of the ABIs outlined above are validated for use in a routine AM/PM scheme for surplus, unweaned calves. Indeed, validity is arguably the most important consideration, such that the chosen ABI reflect calf welfare on farm, during transport and pre-slaughter as intended [(31, 140)]. It is also important that the ABIs are repeatable in terms of producing the same result for repeated observations of the same animal by the same and different observers. For example, Teixeira et al. (141) found a significant effect of meat inspector shift on reasons for carcass condemnation. Standardized recording systems can help such as the calf health scoring chart developed by The University of Madison-Wisconsin School of Veterinary Medicine (https://www.vetmed.wisc.edu/fapm/svm-dairy-apps/calf-health-scorer-chs/) which several authors employed for calves at veal auctions [e.g., (67, 84)]. The associated APP allows scoring of clinical signs on a four-point scale related to respiratory disease (142), diarrhea (143), and navel and joint inflammation (144). As all of these are relevant to slaughter calves, it could be modified for AM use.

Importantly, the ABIs also need to produce consistently reliable results across observations of different animals and they need to be feasible in terms of speed and cost (145). Clearly, they should not compromise normal operating procedures and in this respect consultation with stakeholders is critical (146). Other practical considerations such as the degree of automation of the abattoir, the line speed, and the amount of variation in the training and experience of the veterinary inspectors are also important.

### Feedback of Data

Ultimately, data collected on ABI relevant to calf welfare in the abattoir whether as part of routine veterinary surveillance or by more comprehensive welfare audits should be provided to farmers so that they can benchmark themselves against their peers and to inform animal management plans (33). Toward this end, education of farmers on calf care was identified as a critical finding of a recent needs analysis of male dairy calf marketing (106). By providing farmers with better access to their own data, animal welfare is improved (147, 148). The latter authors found that benchmarking encouraged farmers to make changes to their calf management practices by identifying areas needing attention and promoting discussion about best practices. However, abattoir-based findings on calf welfare are also of interest to cattle veterinarians who play a central role in improving youngstock management on-farm through effective communication of best practice recommendations (149)]. In addition, one needs to be cognisant of the possible divergence in opinion between farmers and stakeholders regarding prioritization of animal welfare issues (150). Other relevant stakeholders include national governmental and non-governmental public-good animal health and welfare organizations, quality assurance schemes and/or retailer groups. This raises issues about confidentiality and data sharing which can be contentious. Ultimately, national benchmark data on findings need to be generated and disseminated to demonstrate temporal and regional trends in progress toward improved ABIs included in the AM/PM calf welfare scheme.

## Conclusions

The most important outcome from this review is the proposal, for the first time, of an abattoir-based AM/PM calf welfare scheme. This scheme in conjunction with a positive feedback loop would ensure critical calf welfare-associated information is communicated to on-farm decision makers and off-farm key stakeholders with the common purpose of improving the welfare of surplus dairy calves destined for early slaughter. This proposal is set within the unique concerns regarding the welfare of surplus dairy calves internationally and the context of existing similar schemes in other species. Such schemes can be used to identify and remediate farms with poor animal welfare and provide real-time and trending industry benchmark data on animal welfare, critical to quality assurance schemes. An abattoir-based AM/PM calf welfare scheme will ultimately provide the evidence-base to protect and enhance dairy industry's reputation amongst increasingly animal welfare-conscious consumers.

## Ethics Statement

Written informed consent was obtained from the individual(s) for the publication of any potentially identifiable images or data included in this article.

## Author Contributions

LB and JM participated equally in the conception, writing and refining of this paper.

## Conflict of Interest

The authors declare that the research was conducted in the absence of any commercial or financial relationships that could be construed as a potential conflict of interest.
